# Routine cognitive screening in older patients admitted to acute medicine: abbreviated mental test score (AMTS) and subjective memory complaint versus Montreal Cognitive Assessment and IQCODE

**DOI:** 10.1093/ageing/afv134

**Published:** 2015-10-13

**Authors:** S. T. Pendlebury, S. P. Klaus, M. Mather, M. de Brito, R. M. Wharton

**Affiliations:** 1 Stroke Prevention Research Unit, Nuffield Department of Clinical Neurosciences, University of Oxford, UK; 2 NIHR Oxford Biomedical Research Centre, John Radcliffe Hospital, Oxford, UK; 3 Departments of Medicine and Geratology, John Radcliffe Hospital, Oxford, UK

**Keywords:** AMTS, subjective memory complaint, Montreal Cognitive Assessment, IQCODE, cognitive screening, older people

## Abstract

**Introduction:** routine cognitive screening for in-patients aged ≥75 years is recommended, but there is uncertainty around how this should be operationalised. We therefore determined the feasibility and reliability of the Abbreviated mental test score (AMTS/10) and its relationship to subjective memory complaint, Montreal Cognitive Assessment (MoCA/30) and informant report in unselected older admissions.

**Methods:** consecutive acute general medicine patients aged ≥75 years admitted over 10 weeks (March–May 2013) had AMTS and a question regarding subjective memory complaint (if no known dementia/delirium). At ≥72 h, the 30-point Montreal Cognitive Assessment (MoCA) and Informant Questionnaire for Cognitive Decline in the Elderly (IQCODE) were done. Cognitive impairment was defined as AMTS < 9 or MoCA < 26 (mild impairment) and MoCA < 20 (moderate/severe impairment) or IQCODE ≥ 3.6.

**Results:** among 264 patients (mean age/SD = 84.3/5.6 years, 117 (44%) male), 228 (86%) were testable with AMTS. 49/50 (98%) testable patients with dementia/delirium had low AMTS compared with 79/199 (44%) of those without (*P* < 0.001). Subjective memory complaint agreed poorly with objective cognitive deficit (39% denying a memory problem had AMTS < 9 (kappa = 0.134, *P* = 0.086)) as did informant report (kappa = 0.18, *P* = 0.15). In contrast, correlation between AMTS and MoCA was strong (*R*^2^ = 0.59, *P* < 0.001) with good agreement between AMTS < 9 and MoCA < 20 (kappa = 0.50, *P* < 0.01), although 85% of patients with normal AMTS had MoCA < 26.

**Conclusions:** the AMTS was feasible and valid in older acute medicine patients agreeing well with the MoCA albeit with a ceiling effect. Objective cognitive deficits were prevalent in patients without known dementia or delirium but were not reliably identified by subjective cognitive complaint or informant report.

## Introduction

Up to one half of the in-patient population of the average general hospital is aged over 65 years and many have co-morbid cognitive impairment associated with high care needs and poor outcomes including increased mortality, complications and institutionalisation [1, 2]. Dementia (often previously undiagnosed) and delirium are prevalent, and decrements in cognitive function may also occur in acute illness in the absence of overt delirium [3–6]. However, services in the general hospital have often failed to adapt to the increasing numbers of frail patients with multiple co-morbidities [1, 2], and cognitive impairment is often not recognised by staff because of a tendency to focus on physical rather than mental health [4, 7].

Routine cognitive screening for older people admitted to the general hospital is therefore recommended (www.england.nhs.uk/wp-content/uploads/2013/02/cquin-guidance.pdf; https://www.rcplondon.ac.uk/sites/default/…/concise-delirium-2006.pdf; www.alzheimers.org.uk/site/scripts/download.php?fileID=1661) but needs to be feasible and pragmatic in view of resource constraints and patient acceptability. A brief quantitative and objective measure of cognitive function, at the point of admission, will provide a baseline record including in those with known dementia, facilitate delirium diagnosis and inform clinical decision-making particularly around early involvement of families and consent processes. The abbreviated mental test score (AMTS) [8] is recommended as a brief pragmatic test of cognitive function in the general hospital (www.england.nhs.uk/wp-content/uploads/2013/02/cquin-guidance.pdf; https://www.rcplondon.ac.uk/sites/default/…/concise-delirium-2006.pdf; www.alzheimers.org.uk/site/scripts/download.php?fileID=1661), but there are few contemporary data particularly in the hyper-acute setting.

We therefore determined the feasibility and validity of the AMTS performed at the point of admission to the general hospital in a consecutive cohort of patients aged ≥75 years admitted to acute general (internal) medicine. Specifically, we aimed to determine (i) the rates and reasons for untestability using the AMTS, (ii) whether subjective memory complaint agreed with objective cognitive deficit as defined by the AMTS and (iii) whether the AMTS identified objective cognitive deficit detected on the more detailed Montreal Cognitive Assessment (MoCA) [9] and an informant-based test for pre-morbid cognitive function, the informant questionnaire for cognitive decline in the elderly (IQCODE) [10].

## Methods

The Oxford University Hospitals Trust provides services for all acute medicine patients in a population of ∼500,000 and runs an unselected medical admissions system irrespective of age, with the majority of patients remaining under the admitting team. In a prospective observational audit, consecutive admissions to a single team over a 10-week period, March–May 2013, were admitted using a structured clerking proforma. The proforma included a cognitive screen on the front page (Supplementary data, Appendix S1, available in *Age and Ageing* online) completed by the admitting team with the AMTS, confusion assessment method (CAM) [11], documentation of pre-admission dementia and of prevalent delirium and a single question to establish the presence of subjective memory complaint. The memory question was reproduced as published in UK national guidelines for dementia screening in older patients with unplanned admission to the general hospital (www.england.nhs.uk/wp-content/uploads/2013/02/cquin-guidance.pdf): ‘have you/has the patient been more forgetful in the past 12 months to the extent that it has significantly affected your/their daily life’ and was only directed to patients without known dementia or delirium. Reasons for not being tested were prospectively recorded as per the proforma. All junior medical staff at OUH are trained by STP (a consultant physician dually accredited in acute general (internal) medicine and geriatrics with expertise in cognitive impairment) in the use of the AMTS and CAM and to complete the cognitive screen on admission for patients aged ≥75 years.

At ≥72 h, repeat AMTS was performed together with the 30-point MoCA [9] and 16-item Informant questionnaire for cognitive decline in the elderly (IQCODE) [10] by S.P.K. and M.M., medical students trained by S.T.P. Demographic data and length of stay in the acute hospital were taken from the patient record.

The study was undertaken to inform future service development and was approved by the Divisional Management and registered with the Oxford University Hospitals Audit Team (audit registration (datix) number 2117).

### Statistical analyses

The UK guidelines for dementia screening in the general hospital recommend using an AMTS cut-off of <9 (www.england.nhs.uk/wp-content/uploads/2013/02/cquin-guidance.pdf) to prompt specialist assessment for possible dementia. We also examined the cut-off of <8 since this is more commonly cited in the literature [12]. For the MoCA, cut-offs of <26 for mild and <20 for moderate/severe cognitive impairment were used as described in the literature [9, 13], and IQCODE ≥ 3.6 was used to indicate pre-admission dementia [10].

Mean and median AMTS and MoCA scores were calculated for the cohort overall and for patients with versus without a cognitive diagnosis (dementia and or delirium on admission). Comparisons were made using *t*-test for continuous variables and *χ*^2^ for categorical variables. Agreement levels were calculated using kappa statistic.

## Results

Among 264 patients (mean age/SD 84.3/5.6 years, range 75–101 years, median (IQR) 84 (80–88) years, 117 (44%) male), 228 (86%) overall, 178/199 (89%) without and 50/65 (77%) with dementia/delirium were testable with the AMTS. The 36 untestable patients were older (86.0/5.9 versus 84.3/5.6 years), more often male (18 (51%) versus 83 (44%), and had more dementia/delirium (15 (42%) versus 50 (22%)). The reasons for untestability were being unwell (*n* = 6), dysphasic (*n* = 9), reduced conscious level/drowsiness (*n* = 8), severe confusion/agitation (*n* = 6), fatigue (*n* = 2), lack of English (*n* = 2) and other (*n* = 3).

**Figure 1.**
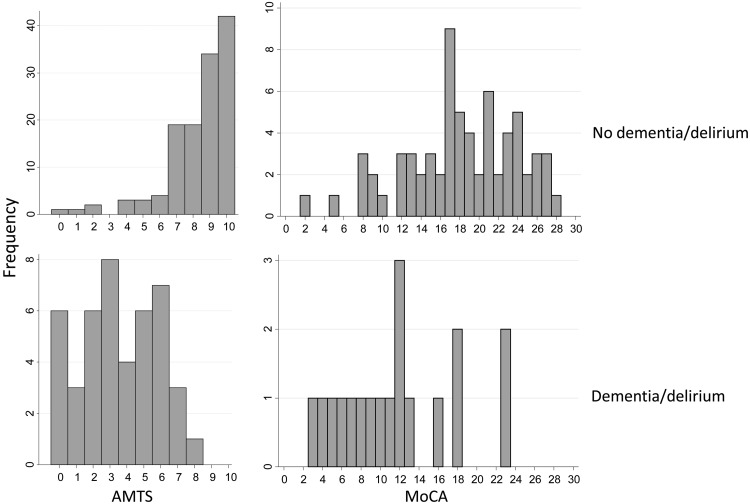
Histograms showing the distribution of AMTS scores (left) and MoCA scores (right) in patients without (top) versus with (bottom) dementia or delirium.

In the 228 testable patients, mean/SD AMTS was 7.2/2.8 with median (IQR) 8 (6–10). AMTS was significantly lower in those with versus without dementia/delirium (mean/SD AMTS 3.8/2.5 versus 8.2/2.0, median (IQR) 4 (2–6) versus 9 (7–10), *P* < 0.001 and scores were skewed towards higher values in those with versus without dementia/delirium (Figure 1)).

Objective cognitive deficit was present in 101 (44%, AMTS < 8) and 128 (56%, AMTS < 9) of testable patients overall. All but one patient (49/50 (98%)) with dementia/delirium had AMTS < 9 and all but two (48/50 (96%)) had AMTS < 8, Given that 15 untestable patients had dementia/delirium, a total of 101 + 15 = 115 (44%, AMTS < 8) and 128 + 15 = 143 (54%, AMTS < 9) in the cohort overall had objective cognitive deficit. In testable patients without dementia/delirium, rates of objective cognitive deficit were lower but remained substantial at 53/178 (30%, AMTS < 8) and 79/178 (44%, AMTS < 9).

One hundred and twenty-six tested patients without dementia/delirium were asked the memory question. Only 26 (21%) had subjective memory complaint (answered ‘yes’ to the memory question), and overall agreement between objective cognitive deficit and subjective memory complaint was poor (kappa = 0.134, *P* = 0.086 for AMTS < 9; kappa = 0.109, *P* = 0.21 for AMTS < 8): of the 100 denying a memory problem, 39 had AMTS < 9 and 26 had AMTS < 8 and 11/26 (42%) with subjective memory complaint had AMTS ≥ 9 and 16 (62%) had AMTS ≥ 8. The probability of having objective impairment for those with versus without subjective memory complaint was 10/26 versus 26/100, OR = 1.77, 95% CI 0.72–4.41, *P* = 0.21 for AMTS < 8 and 15/26 versus 39/100, OR = 2.13, 0.89–5.12, *P* = 0.09 for AMTS < 9.

**Figure 2.**
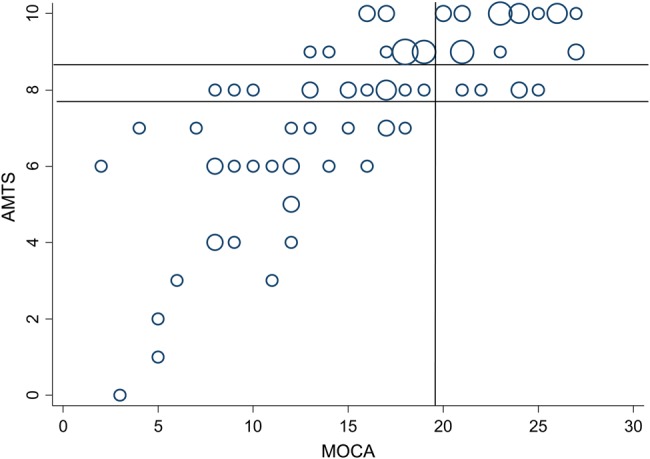
Bubble plot showing the strong correlation between repeat AMTS and MoCA scores (*R*^2^ = 0.59, *P* < 0.001). Lines mark the cut-offs for AMTS < 9 and AMTS < 8 and MoCA < 20.

At ≥72 h, 100 (63%) patients had repeat AMTS, 91 (57%) had MoCA and 65 (41%) had the IQCODE. Objective cognitive deficits were present in a similar proportion of testable patients using repeat AMTS as seen on admission with 40 (40%, AMTS < 8) and 60 (60%, AMTS < 9, Table 1). Mean/SD MoCA was 16.8/6.3 with lower scores in those with versus without dementia/delirium: mean/SD MoCA 11.7/6.0 versus 18/5.8, *P* < 0.001, and scores were normally distributed in both groups (Table 1, Figure 1). The repeat AMTS and the MoCA were highly correlated (Figure 2, *R*^2^ = 0.59, *P* < 0.001, and agreement between the AMTS and MoCA for objective cognitive deficit was good (kappa = 0.39, *P* < 0.01 for AMTS < 8 and Kappa = 0.50, *P* < 0.01 for AMTS < 9 and MoCA < 20). However, although the AMTS had good specificity for MoCA-defined cognitive impairment (all 29 patients with AMTS < 8 had MoCA < 20), sensitivity was relatively less good with a ceiling effect: the majority of patients with normal AMTS scores (28/33, AMTS ≥ 9 and 42/47, AMTS ≥ 8) had MoCA < 26 and one-third to a half had MoCA < 20 (12/33, AMTS ≥ 9 and 24/47, AMTS ≥ 8, Figure 2). There was very poor agreement between subjective memory complaint and objective deficit defined by the MoCA (kappa = 0.008, *P* = 0.88 for MoCA < 26 and kappa = 0.044, *P* = 0.69 for MoCA < 20).

**Table 1.** Demographics and cognitive data for all patients and separately for those with versus without dementia/delirium

	All, *n* = 264	Delirium/Dementia, *n* = 65	No delirium/dementia, *n* = 199	*P*
Age/SD mean	84.3/5.6	85.8/5.2	83.8/5.6	0.014
Age median (IQR), range	84 (80–88), 75–101	87 (82–90), 75–95	83.0 (79–88), 75–101	
Male	117 (44)	23 (35)	94 (47)	0.095
AMTS done	228 (86)	50 (77)	178 (89)	0.011
Median AMTS (IQR)		4 (2–6)	9 (7–10)	<0.001
Mean/SD AMTS		3.8/2.5	8.2/2.0	
AMTS < 8	101 (44)	48 (96)	53 (30)	<0.001
AMTS < 9	128 (56)	49 (98)	79 (44)	<0.001
Memory question done		N/A	128 (68)	
Subjective memory complaint present			27/128 (21)	
ReAMTS done (*n*)	100	23	77	
Median ReAMTS (IQR)		5 (3–7)	8 (7–10)	
Mean/SD ReAMTS		4.9/2.6	8.0/2.2	
ReAMTS < 8	41 (41)	21 (91)	20 (26)	
ReAMTS < 9	60 (60)	21 (91)	39 (51)	
MoCA done (*n*)		18	73	
Median MoCA (IQR)		11.5 (7–16)	18 (15–23)	<0.001
Mean/SD MoCA		11.7/6.0	18.0/5.8	
MoCA < 26	84 (92)	18/18 (100)	66/73 (90)	0.171
MoCA < 20	59 (65)	16/18 (89)	43/73 (59)	0.017
IQ code done (*n*)		11	54	
IQ code ≥3.6	27 (42)	8/11 (73)	19/54 (35)	0.021

Numbers are *n* (%) unless otherwise specified.

Of the 65 patients with IQCODE, 11 had dementia/delirium among whom all 4 patients with dementia and 6/9 with delirium had IQCODE ≥ 3.6. In the remaining 54 patients without dementia/delirium, 19/54 (35%) had abnormal IQCODE. There was a non-significant trend (*P* = 0.11) to agreement between abnormal IQCODE and low AMTS (both on admission and at >72 h) but in the 34 with normal IQCODE who had AMTS, 12 had AMTS < 8 and 15 had AMTS < 9. Conversely, in the 23 with abnormal IQCODE, 10 had AMTS ≥ 8 and 5 had AMTS ≥ 9. 6/24 without subjective memory complaint had abnormal IQCODE.

## Discussion

The AMTS was feasible to perform routinely in consecutive older patients admitted as an emergency to the acute medicine service of the general hospital. Less than one-fifth of patients were untestable overall, most commonly because of severe illness, reduced conscious level or dysphasia. Rates of objective cognitive impairment were high affecting around half the cohort overall and over a third of those without known dementia/delirium in whom subjective cognitive complaint agreed poorly with objective cognitive deficit. In contrast, agreement between the AMTS and MoCA for objective cognitive impairment was good, although the AMTS showed a ceiling effect being insensitive to milder impairment. There was a trend towards greater likelihood of low AMTS score with informant-defined pre-morbid cognitive decline but significant numbers of patients had low cognitive scores in the absence of prior informant-reported deficits.

The current study demonstrates that the AMTS is applicable to the vast majority of older patients at the point of admission and remains a practical and useful test for detecting cognitive impairment despite having been developed some decades ago [8, 14, 15]. The brevity of the AMTS is a significant advantage in busy clinical environments where routine administration of longer tests is impractical: in a previous study, we found the AMTS to be more feasible than the MMSE [16] being relatively easier for patients with hearing or vision difficulties and not requiring a motor response [17].

Our data quantify the high prevalence of cognitive disorders among the older in-patient general hospital population and specifically highlight the substantial rates (30–44%, depending on cut-off used) of objective cognitive deficits in patients without a known dementia/delirium. Routine screening is thus required since clinicians are poor at estimating cognitive function in the absence of an objective test [18]. Cognitive testing also facilitates delirium diagnosis (https://www.rcplondon.ac.uk/sites/default/…/concise-delirium-2006.pdf) [14]: the widely recommended CAM [4, 11] (https://www.rcplondon.ac.uk/sites/default/…/concise-delirium-2006.pdf) was designed to be used with an objective measure of cognitive function. Routine AMTS is advised for hip fracture patients in the UK in recognition of high rates of cognitive impairment and the impact on patient management including consent procedures (https://www.nice.org.uk/guidance/cmg46/chapter/33-quality-measures#332-the-best-practice-tariff).

Our findings suggest that subjective memory complaint determined via the memory question in the acute hospital setting is an unreliable indicator of objective cognitive deficit. Only a fifth of patients without cognitive diagnosis had subjective complaint, a rate much lower than was obtained using objective testing, and many patients who denied memory problems had significant objective deficits and vice versa. Other studies from a wide range of settings including large-scale epidemiological volunteer cohorts [19–22] and disease-based cohorts have shown that subjective complaints do not reliably identify objective deficits or predict likelihood of future objective decline. The current UK recommendation to find older hospitalised patients at risk of dementia through use of the memory question is thus likely to miss many patients at risk (www.england.nhs.uk/wp-content/uploads/2013/02/cquin-guidance.pdf).

The AMTS appeared valid for detecting cognitive impairment as defined by the more detailed and lengthy MoCA [9]. The MoCA was initially developed to detect mild cognitive impairment (MCI) in the memory clinic setting and covers a broader range of cognitive domains than the previously widely used MMSE. The MOCA has been validated for use in acute and chronic conditions including cerebrovascular disease [22] and Parkinson's disease [23] and has good sensitivity and specificity for multiple domain MCI/dementia at cut-offs around 20–22 and is very sensitive but less specific for single domain MCI at cut-offs of <26 [13, 24]. Our findings showed that the AMTS appeared to have a ceiling effect relative to the MoCA: many patients with low MoCA (<20) were nevertheless able to achieve normal AMTS scores. However, patients with low AMTS never had MoCA > 20, demonstrating the specificity of a low AMTS score for cognitive impairment.

Around 40% of patients with IQCODE at 72 h had significant pre-morbid decline according to informant report of whom a minority were able to achieve normal AMTS scores, in keeping with the previously discussed ceiling effect. Conversely, many patients without significant pre-morbid decline nevertheless had objective deficits in the acute hospital setting. This is not surprising given the known impact of acute illness and hospitalisation on cognition particularly in frail older patients and confirms that pre-morbid function cannot be assumed to be maintained in hospital [1, 2, 7].

Strengths of our study include the generalisability of our findings resulting from inclusion of a consecutive cohort of all admitted patients aged ≥75 years and the careful documentation of rates and reasons for untestability with the AMTS. There are also some limitations. We were not able to obtain memory question answers on all patients completing the AMTS since the admitting teams did not always complete this question. Not all patients remaining in hospital at 72 h had the extended cognitive examination including the MoCA and IQCODE owing to logistical difficulties in the acute care setting including illness severity and the need for complex investigations and in locating relatives.

In conclusion, our data show that the AMTS is applicable to the majority of older patients with emergency admission to the general hospital. Rates of AMTS-defined objective cognitive deficits were high even in those without a known previous cognitive diagnosis and agreed well with MoCA-defined impairment but not with subjective memory complaints. Routine cognitive screening using the AMTS will provide an objective baseline measure including in known dementia and will facilitate delirium diagnosis, inform clinical decision-making and highlight patients at risk of dementia for further evaluation in primary care.

**Key points**

- AMTS agrees well with MoCA in acute medicine patients.
- Subjective cognitive complaint agrees poorly with objective deficits in the context of acute illness.
- Informant report agrees poorly with objective deficits in the context of acute illness.

## Supplementary data

Supplementary data mentioned in the text are available to subscribers in *Age and Ageing* online.

## Conflicts of interest

None declared.

## Funding

S.T.P. is supported by the Oxford NIHR Biomedical Research Centre. R.M.W. is supported by the Wellcome Trust.

## Supplementary Material

Supplementary Data
