# Supplementary material for: Routine cognitive screening in older patients admitted to acute medicine: abbreviated mental test score (AMTS) and subjective memory complaint versus Montreal Cognitive Assessment and IQCODE
Source: Age Ageing. 2015 Oct 13;44(6):1000–5. doi: 10.1093/ageing/afv134 (PMC4621235; doi:10.1093/ageing/afv134)
Supplement: Supplementary Data [file supp_44_6_1000__index.html]

Routine cognitive screening in older patients admitted to acute medicine: abbreviated mental test score (AMTS) and subjective memory complaint versus Montreal Cognitive Assessment and IQCODE — Supplementary Data 

# Routine cognitive screening in older patients admitted to acute medicine: abbreviated mental test score (AMTS) and subjective memory complaint versus Montreal Cognitive Assessment and IQCODE

## Supplementary Data

Supplementary Data

- Supplementary Data - Docx file
