# Supplementary figures and images for: Routine cognitive screening in older patients admitted to acute medicine: abbreviated mental test score (AMTS) and subjective memory complaint versus Montreal Cognitive Assessment and IQCODE
Source: Age Ageing. 2015 Oct 13;44(6):1000–5. doi: 10.1093/ageing/afv134 (PMC4621235; doi:10.1093/ageing/afv134)

**SUPPLEMENTARY DATA**

Appendix figure

**
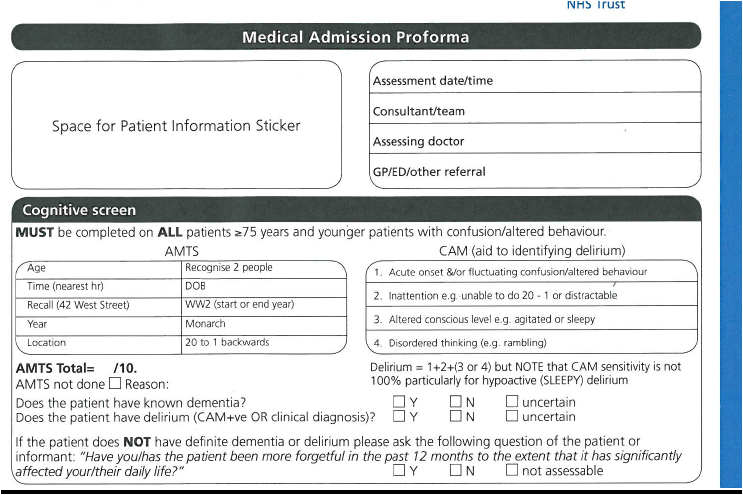
**

Supplement: Supplementary Data [file supp_afv134_afv134supp.docx]
